# Estudio bioquímico de neuroblastoma congénito

**DOI:** 10.1515/almed-2022-0049

**Published:** 2022-12-15

**Authors:** Cristina Montero Domínguez, Alicia Ortiz Temprado, Laura Martínez Figueras, Alba Guillamón Seoane, Miguel Fernández Ruano

**Affiliations:** Servicio de Bioquímica Clínica, Hospital General Universitario Gregorio Marañón, Madrid, España

**Keywords:** ácido homovanílico, ácido vanilmandélico, catecolaminas, índice catecolamina/creatinina, metanefrinas fraccionadas, neuroblastoma congénito

## Abstract

**Objetivos:**

La incidencia de neuroblastoma congénito se ha incrementado en los últimos años. El propósito de este artículo es presentar las características clínicas y bioquímicas de neuroblastomas congénitos diagnosticados en nuestro centro.

**Caso clínico:**

Estudio de tres casos de neuroblastoma congénito diagnosticados en nuestro hospital. En dos casos el diagnóstico fue prenatal y en el otro fue detectado en el período neonatal inmediato. Los tres casos presentaron localización abdominal y valores de catecolaminas o sus metabolitos en orina de micción única elevados. Dos tumores se clasificaron como estadio M y uno como estadio L2. En ninguno de los pacientes estudiados se encontró amplificación del oncogén *N-MYC* ni presentaron características histopatológicas desfavorables. Se realizó exéresis del tumor en dos pacientes, y los tres recibieron quimioterapia.

**Conclusiones:**

La medición de catecolaminas y sus metabolitos en orina es una parte fundamental del diagnóstico de neuroblastoma. En aquellos casos en los que no se puede recoger muestras de orina de 24 horas, se puede utilizar orina de micción única calculando el índice en función de la creatinina excretada.

## Introducción

El neuroblastoma (NBL) es el tumor sólido extracraneal más frecuente en la infancia con una incidencia de 10 casos por millón de niños menores de 15 años. Representa el 7% de todos los cánceres pediátricos y es la causa del 15% de muertes por procesos oncológicos en la infancia [1]. Se origina a partir de células de la cresta neural y puede localizarse en cualquier parte de la cadena nerviosa simpática neuroectodérmica. La sintomatología depende de la localización del tumor primario y la presencia de síndromes paraneoplásicos o metástasis. El 90% secretan catecolaminas, por lo que para su diagnóstico se utilizan tanto marcadores bioquímicos (determinación de catecolaminas y sus metabolitos) como pruebas de imagen y estudios histológicos [1, 2].

Se considera NBL congénito a aquel que se detecta durante el embarazo o en los 28 días posteriores al nacimiento, representando el 5% de todos los NBL [2]. En el caso de los NBL diagnosticados prenatalmente más del 90% tienen su origen en la médula suprarrenal [3, 4], por lo que una masa sólida suprarrenal en ecografía prenatal sugiere el diagnóstico de NBL congénito. En estos casos es necesario hacer el diagnóstico diferencial con otras alteraciones como hidronefrosis, hemorragia suprarrenal y secuestros pulmonares extralobares infradiafragmáticos [2, 3]. Debido al uso creciente de la ecografía prenatal, la detección de NBL congénito se ha incrementado en los últimos años, cobrando así mayor importancia el conocimiento sobre su manejo en lactantes.

## Caso clínico

En este trabajo se analizan tres casos de NBL congénito diagnosticados en nuestro centro entre 2019–2020. En la Tabla 1 se recoge un resumen de estos casos.

**Tabla 1:** Resumen de los casos de NBL congénito.

Caso	Edad al diagnóstico	Localización tumor primario	Localización metástasis	Catecolaminas o metabolitos elevados	Amplificación *N-MYC*	Histopatología	Estadio al diagnóstico	Tratamiento
A	37 SG	Adrenal	Ósea y hepática	NA/creatinina NMN/creatinina HVA/creatinina VMA/creatinina	No	Favorable	M de riesgo intermedio	Cirugía y quimioterapia
B	1 día de vida	Adrenal	–	NMN/creatinina HVA/creatinina VMA/creatinina	No	Favorable	L2 de bajo riesgo	Quimioterapia
C	34 SG	Adrenal	Hepáticas, retroperitoneales, subcutáneas y musculares	NMN/creatinina HVA/creatinina VMA/creatinina	No	Favorable	M de riesgo intermedio	Quimioterapia y cirugía

SG, semana de gestación; NA, noradrenalina; NMN, normatenefrina; VMA, ácido vanilmandélico; HVA, ácido homovanílico.

### Caso A

Gestante de 29 años (3 gestaciones, 0 abortos) en la que se objetivó por ecografía a las 37 semanas de gestación una masa de localización suprarrenal en el feto. Las imágenes de resonancia magnética (RM) confirmaron la presencia de una masa tumoral de 2,8 × 2,8 × 3,6 cm en región suprarrenal derecha sugestiva de NBL. El parto fue eutócico a las 40 + 6 semanas de gestación, varón con Apgar 9/10 y peso 3,360 g. La ecografía postnatal confirmó la presencia de una masa de 5 × 3,3 × 3,6 cm.

**Figura 1:**
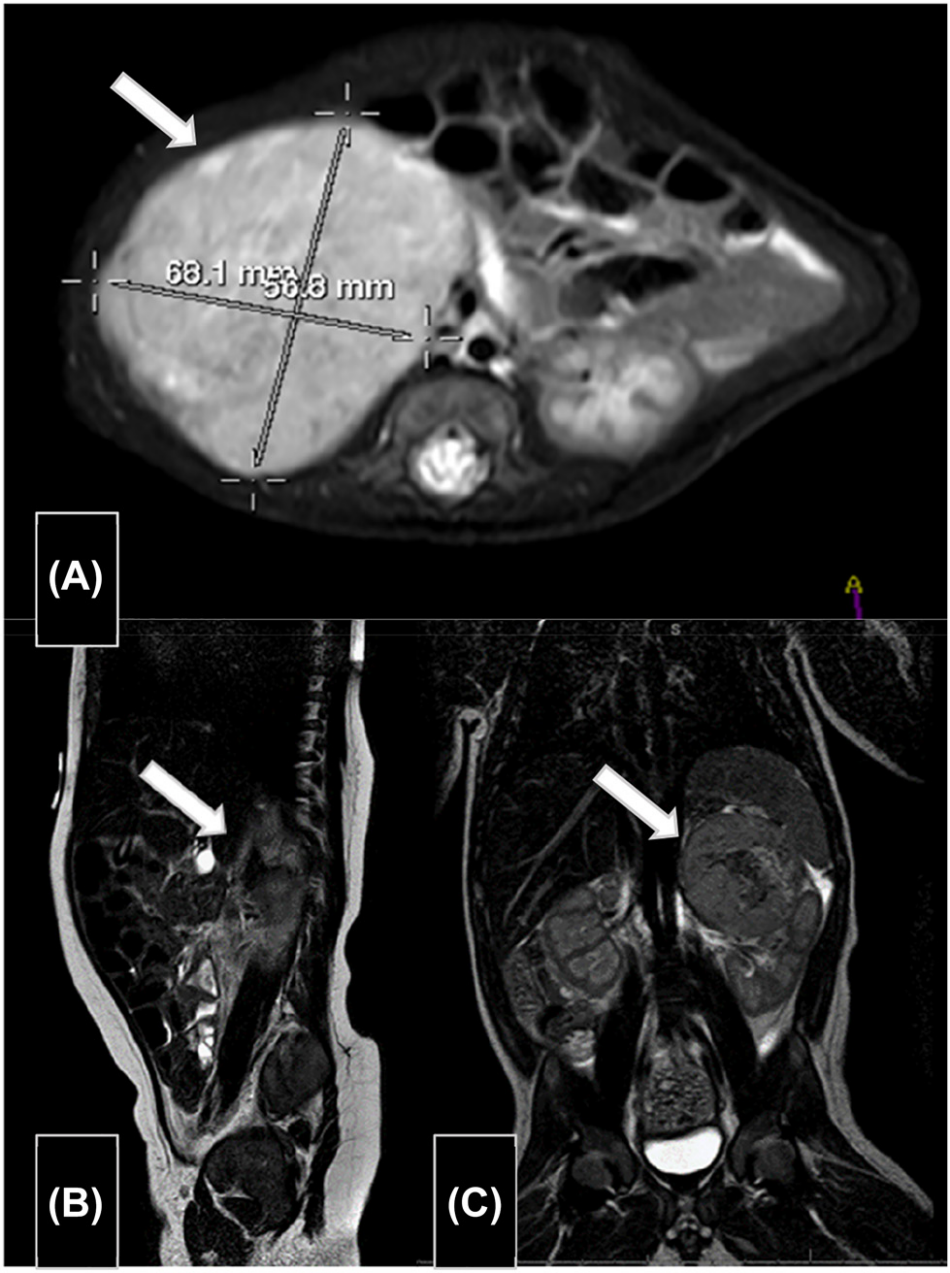
Resonancias magnéticas abdominales. (A) Caso A: Masa de 6,8 × 5,7 × 7,6 cm en la región suprarrenal derecha. Contacta con la vena cava inferior y la vena y arteria renal derecha, así como el riñón derecho al que deforma y desplaza inferiormente y el lóbulo hepático derecho. (B) Caso B: Masa sólida en área suprarrenal derecha de 3,6 × 2,6 × 5,1 cm. (C) Caso C: Masa suprarrenal izquierda de 4,4 × 4,2 × 4,0 cm.

Al nacimiento se recogieron muestras de orina de micción única seriadas durante tres días para la determinación de catecolaminas y sus metabolitos mediante cromatografía líquida de alta resolución (HPLC) en un equipo Agilent^®^ 1100 (Agilent Technologies, Santa Clara, Estados Unidos) utilizando kits comerciales de Recipe^®^ (RECIPE Chemicals + Instruments GmbH, Múnich, Alemania). Se obtuvieron valores elevados para las ratios noradrenalina (NA)/creatinina, normetanefrina (NMN)/creatinina, ácido homovanílico (HVA)/creatinina y ácido vanilmandélico (VMA)/creatinina (Tabla 2). Al mes de vida se realizó una RM abdominal donde se observó crecimiento de la masa suprarrenal derecha, de 6,8 × 5,7 × 7,6 cm (Figura 1A). La gammagrafía con ^131^I-metaiodo-benzil-guanidina (MIBG) fue compatible con la existencia de tejido cromafín patológico en la masa, con afectación ósea multifocal y hepática.

**Tabla 2:** Análisis bioquímico por HPLC de catecolaminas y sus metabolitos.

	Intervalo de referencia	Caso A	Caso B	Caso C
A, nmol/mmol creatinina	<42,6 [10]	18,3 (10,1)	–	28,0 (13,0)^b^
NA, nmol/mmol creatinina	<182 [10]	**802, 0 (163,0)**	–	76,7 (21,9)^b^
DA, nmol/mmol creatinina	<1975 [10]	809 (40,8)	–	866,6 (6,3)^b^
MN, nmol/mmol creatinina	50–400 [11]	235,1 (83)	161,8 (81,3)	83,2 (36,9)
NMN, nmol/mmol creatinina	590–1520 [11]	**21745,6 (7335,4)**	**6658,9 (4627,5)**	**1971,3 (119,6)**
3-MT, nmol/mmol creatinina	No disponible	2570,6 (647,0)	975,0 (722,2)	514,4 (94,1)
VMA, µmol/mmol creatinina	<10,8 [10]	**115,2 (9,5)**	**35,6 (14,9)**	**43,6 (5,3)** ^**b**^
HVA, µmol/mmol creatinina	<21,7 [10]	**144,1 (44,9)**	**36,4** ^**a**^	**60,9 (3,7)** ^**b**^

Los resultados de los casos se expresan como media (desviación estándar), destacándose en negrita aquellos casos en los que los valores son superiores al intervalo de referencia. A, adrenalina; NA, noradrenalina; DA, dopamina; MN, metanefrina; NMN, normatenefrina; 3-MT, 3-metoxitiramina; VMA, ácido vanilmandélico; HVA, ácido homovanílico. ^a^Solo se dispone de datos de un día. ^b^Se dispone de datos de dos días.

Se inició tratamiento alfabloqueante y betabloqueante debido a las crisis hipertensivas que sufría el paciente, para llevar a cabo cirugía excisional de la masa, que se realizó por laparotomía a los dos meses de vida. El estudio anatomopatológico informó de NBL pobremente diferenciado con índice de mitosis-cariorrexis (MKI) bajo. En el estudio genético no se detectó amplificación del oncogén *N-MYC* ni deleción del cromosoma 11q. Se clasificó como un estadio M de riesgo intermedio (International Neuroblastoma Risk Group Staging System [INRGSS]). Posteriormente, el paciente recibió tratamiento quimioterápico (etopósido/carboplatino).

### Caso B

Recién nacido, con diagnóstico prenatal de síndrome de Noonan, con Apgar 8/10 y peso 3,050 g. Se identificó en ecografía una masa en región suprarrenal derecha sugestiva de NBL congénito. El análisis en orina de micción única mostró valores elevados de los metabolitos de catecolaminas, especialmente para la ratio NMN/creatinina (Tabla 2). La RM confirmó la presencia de una masa sólida en área suprarrenal derecha de 3,6 × 2,6 × 5,1 cm compatible con la sospecha diagnóstica (Figura 1B). La gammagrafía con ^131^I-MIBG mostró la presencia de tejido cromafín patológico a nivel de glándula suprarrenal derecha, sin afectación en otras localizaciones.

El estudio anatomopatológico informó de NBL pobremente diferenciado con MKI bajo. El estudio genético reflejó ausencia de amplificación *N-MYC* y 11q no delecionado. Se clasificó como un estadio L2 de bajo riesgo (INRGSS), recibiendo tratamiento quimioterápico (etopósido/carboplatino).

### Caso C

Gestante de 35 años (3 gestaciones, 2 abortos) en la que se objetivó por ecografía a las 34 semanas de gestación agenesia completa del cuerpo calloso y masa adrenal superior izquierda en el feto. La RM fetal mostró una masa tumoral de 2,9 × 2,3 × 3,2 cm. El parto fue eutócico a las 39 semanas de gestación, recién nacido varón con Apgar 9/10 y peso 3,130 g. La ecografía postnatal evidenció una masa en hipocondrio izquierdo de 5,3 × 3,5 × 4 cm, sugestiva de NBL.

La medición de catecolaminas y sus metabolitos mostró valores ligeramente elevados para las ratios NMN/creatinina, HVA/creatinina y VMA/creatinina en las muestras seriadas de orina de micción única (Tabla 2). A los tres días del nacimiento se realizó una RM cerebral que confirmó el diagnóstico prenatal de agenesia completa de cuerpo calloso y mostró alteraciones malformativas asociadas. A los 11 días de vida la RM abdominal (Figura 1C) confirmó la presencia de NBL con metástasis hepáticas, retroperitoneales, subcutáneas y musculares. El estudio anatomopatológico informó de NBL pobremente indiferenciado con MKI bajo. En el estudio genético no se detectó amplificación del oncogén *N-MYC* ni deleción del cromosoma 11q. Se clasificó como un estadio M de riesgo intermedio (INRGSS).

Al mes de vida se inició tratamiento quimioterápico (etopósido/carboplatino), tras el cual se observó disminución del tamaño de la masa suprarrenal izquierda, así como una respuesta favorable de las lesiones hepáticas, subcutáneas y musculares. A los 4 meses de vida se procedió a la exéresis del tumor.

## Discusión

Actualmente, el NBL se diagnostica mediante análisis de catecolaminas y sus metabolitos en orina, histopatología y técnicas de imagen [5]. Tradicionalmente el análisis más utilizado ha sido la determinación de VMA y HVA, con una sensibilidad de 81,6% y 80,5% respectivamente, obteniéndose los mejores rendimientos en presencia de enfermedad diseminada (sensibilidad del 100%, especificidad del 99,7% y área bajo la curva (AUC) de 1 al combinar la medición de HVA y VMA) [6]. Otros estudios han observado que la combinación de NMN con VMA o HVA mejora el rendimiento diagnóstico, y la inclusión de la 3-metoxitiramina aumentaría la sensibilidad diagnóstica hasta el 95% [7], [8], [9]. En los casos de NBL congénitos publicados los datos de perfil de secreción son escasos, habiéndose descrito valores aumentados de VMA y HVA en orina en aproximadamente el 33–38% de los NBL diagnosticados prenatalmente [3, 4]. En nuestros tres casos, todos los pacientes presentaron al menos una de las magnitudes medidas elevada; esto puede deberse al mayor tamaño de los tumores documentados en este trabajo, a la medición de más metabolitos como la NMN o al hecho de que nuestros resultados fueran corregidos con la excreción de creatinina.

Una de las dificultades para el estudio de los metabolitos de catecolaminas urinarias en pacientes pediátricos es la obtención de muestra de orina de 24 horas. En estos casos se puede utilizar orina de micción única calculando el índice en función de la creatinina excretada. Es necesario tener en cuenta que la excreción de creatinina está influenciada por la masa muscular y por tanto aumenta con el crecimiento, siendo necesario un rango de referencia apropiado para cada edad [10, 11].

El grupo de trabajo International Neuroblastoma Risk Group desarrolló el INRGSS para clasificar este tipo de tumores basándose en las técnicas de imagen previas a la cirugía. Este grupo además identificó otros factores pronósticos principales que contribuyen a la gravedad de la enfermedad: la edad al momento del diagnóstico (>18 meses), la histología y grado de diferenciación y los datos citogenéticos y moleculares [12]. Ninguno de los NBL congénitos de nuestros casos presentaron características histopatológicas desfavorables, amplificación del gen *N-MYC* o aberraciones cromosómicas.

El NBL comprende tumores clínicamente heterogéneos que presentan un amplio espectro de comportamiento. El correcto diagnóstico determinará el tipo de tratamiento que puede variar desde una actitud expectante a quimioterapia intensiva y cirugía [1, 2]. Es necesario realizar un diagnóstico diferencial precoz para el adecuado manejo terapéutico, en el que tiene especial importancia la medición de metabolitos de catecolaminas urinarios.

## Puntos clave

– El NBL congénito es una entidad poco frecuente, pero que debe tenerse en cuenta ante el hallazgo de una masa sólida suprarrenal en ecografía prenatal.
– La sospecha prenatal de NBL permite realizar de manera inmediata tras el nacimiento el estudio bioquímico que conduce al diagnóstico. La determinación de metabolitos de catecolaminas en orina es uno de los métodos más fiables para el diagnóstico.
– La corrección con creatinina y la disponibilidad de valores de referencia ajustados por edad permite el análisis en orina de micción única en aquellos pacientes en los que no es posible recoger orina de 24 horas.
